# Bone marrow derived-mesenchymal stem cells downregulate IL17A dependent IL6/STAT3 signaling pathway in CCl_4_-induced rat liver fibrosis

**DOI:** 10.1371/journal.pone.0206130

**Published:** 2018-10-22

**Authors:** Shimaa Farouk, Salwa Sabet, Fatma A. Abu Zahra, Akmal A. El-Ghor

**Affiliations:** 1 Department of Biology and Biotechnologies, Faculty of Science & Technology, AL-Neelain University, Khartoum, Sudan; 2 Department of Zoology, Faculty of Science, Cairo University, Giza, Egypt; 3 Medical Research Center, Faculty of Medicine, Ain Shams University, Cairo, Egypt; Centro Cardiologico Monzino, ITALY

## Abstract

Therapeutic potential of bone marrow–derived mesenchymal stem cells (BM-MSCs) has been reported in several animal models of liver fibrosis. Interleukin (IL) 17A, IL6 and Stat3 have been described to play crucial roles in chronic liver injury. However, the modulatory effect of MSCs on these markers was controversial in different diseases. BM-MSCs might activate the IL6/STAT3 signaling pathway and promote cell invasion in hepatocellular carcinoma, but the immunomodulatory role of BM-MSCs on IL17A/IL6/STAT3 was not fully elucidated in liver fibrosis. In the present study, we evaluated the capacity of the BM-MSCs in the modulation of cytokines milieu and signal transducers, based on unique inflammatory genes Il17a and Il17f and their receptors Il17rc and their effect on the IL6/STAT3 pathway in CCl_4_-induced liver fibrosis in rats. A single dose of BM-MSCs was administered to the group with induced liver fibrosis, and the genes and proteins of interest were evaluated along six weeks after treatment. Our results showed a significant downregulation of *Il17a*, *Il17ra*, *il17f* and *Il17rc* genes. In accordance, BM-MSCs administration declined IL17, IL2 and IL6 serum proteins and downregulated IL17A and IL17RA proteins in liver tissue. Interestingly, BM-MSCs downregulated both *Stat3* mRNA expression and p-STAT3, while *Stat5a* gene was downregulated and p-STAT5 protein was elevated. Also P-SMAD3 and TGFβR2 proteins were downregulated in response to BM-MSCs treatment. Collectively, we suggest that BM-MSCs might play an immunomodulatory role in the treatment of liver fibrosis through downregulation of IL17A affecting IL6/STAT3 signaling pathway.

## Introduction

Liver fibrosis represents the final common pathway of virtually all chronic liver diseases including viral hepatitis, alcohol or drug abuse and metabolic diseases [1], and thus represents the first step toward a number of often mortal complications of liver disease. An effective treatment for liver fibrosis, aside from liver transplantation, has not been established yet [2]. Therefore, alternative treatments have been considered such as stem cells transplantation as these cells have the ability of self-renewal and differentiation [3].

Mesenchymal stem cells (MSCs) are multipotent adult stem cells that have the ability to differentiate into hepatocyte like cells [4, 5], and have the capacity to secrete a series of cytokines and signaling molecules [6, 7] which can regulate inflammatory responses, stimulate hepatocyte proliferation, and maintain hepatocyte function [8]. In a mouse model of liver failure, the systemic injection of bone marrow or bone marrow-derived MSCs (BM-MSCs) into mice have rescued the disease phenotype [9, 10]. Moreover, anti-fibrotic activities of MSCs have been reported in fibrotic animal models [11, 12].

Several studies have referred to interleukin 17A (IL17A) as a critical key player in liver fibrosis [13, 14]. In different cell types of liver, including hepatic stellate cells (HSCs), IL17 receptors complex IL17RA/IL17RC induce signals that activate intracellular factors such as signal transducer and activator of transcription3 (STAT3) which lead to inflammation and liver fibrosis progression [15, 16]. Activation of STAT3 has been detected in all rodent models of liver injury [17, 18]. Inhibition of the STAT3 pathway in HSCs induces apoptosis [19] and renders cells less susceptible to fibrosis [16, 20]. Interleukin 6 (IL6) stimulates the activation of STAT3 and increases collagen mRNA expression in HSCs, thus accelerating liver fibrosis via Stat3 phosphorylation that in turn activates transforming growth factor beta (TGFβ) signaling cascade through SMAD3 activation [21]. TGFβ when binds to type II TGFβ receptor (TGFβR2), influences activation of Smads dependent pathway [22]. SMAD3 is the predominant mediator of fibrogenic TGFβ downstream signaling and main mediator of fibrogenic response in HSCs [23, 24]. However, the role of STAT3 pathway in liver fibrogenesis appears to be controversial due to the recorded hepatoprotective and proliferative functions of STAT3 [25, 26].

The immunosuppressive effects of MSCs on the inflammation that is associated with hepatic fibrosis are mainly due to MSC-derived secretomes. Hence, MSC-derived soluble factors promote development of anti-inflammatory T-regulatory cells (Tregs) and reduce influx of inflammatory Th17 cells in the inflamed liver [27, 28]. Inhibition of inflammation is one of the therapeutic approaches of liver fibrosis. The IL17A/STAT3 pathway is critical in liver fibrosis [29]. Although the effect of BM-MSCs on IL6/STAT3 pathway was conducted in cancer and has shown that MSCs could promote progression of cancer through activation of IL6/STAT3 pathway [30], the immunomodulatory role of BM-MSCs on IL17A/IL6/STAT3 was not fully elucidated in liver fibrosis. Thus, we investigated the effect of BM-MSCs on IL17A dependent IL6/STAT3 pathway.

## Methods

### Isolation, purification and characterization of bone marrow-derived mesenchymal stem cells (BM-MSCs)

BM-MSCs were prepared from the tibia and femurs of rats as previously described [31]. Whole Bone-Marrow (100±20g) was flushed with Dulbecco’s modified Eagle’s medium (DMEM) supplemented with 10% bovine serum (Gibco-BRL, Grand Island, NY, USA). According to the manufacturer’s instructions of the Ficoll density gradient media (Biochrom, Berlin, Germany), nucleated cells were isolated and resuspended in a complete culture medium supplemented with 1% penicillin-streptomycin (Gibco-BRL, Grand Island, NY, USA) and then were incubated at 37°C in 5% humidified CO_2_ for 12–14 days. When cultures approached 80% confluence, they were washed twice with phosphate buffer saline (PBS) and were trypsinized with 0.25% trypsin in 1 mM EDTA (Gibco-BRL, Grand Island, NY, USA) for 45 min at 37°C under shaking. Cells were centrifuged then resuspended in serum supplemented medium and incubated in 50 cm^2^ culture flask and the submitted cultures were claimed as first-passage cultures. Cells were serially subcultured to passage four and cells at passage three (P3) were stained for 30 min with FITC-conjugated anti-rat CD29, PE-conjugated anti-rat CD90 and PE-conjugated anti-rat CD34 antibodies (Beckman Coulter, Brea, CA, USA). Cell analysis was performed using CYTOMICS FC 500 Flow Cytometer (Beckman Coulter, Brea, CA, USA) and analyzed using CXP Software version 2.2.

### Labeling of MSCs with PKH26

MSCs were collected during the 4th passage and labeled with PKH26 Red Fluorescent Cell Linker kit (Sigma-Aldrich, Saint Louis, Missouri, USA), according to the manufacturer’s instructions. Labeled MSCs were intravenously injected into the tail vein of fifteen rats. Rats were dissected and Liver tissues were examined with a fluorescence microscope to detect and trace the labeled stem cells.

### Animals, CCl_4_ induction and treatment with BM-MSCs

Thirty male Sprague-Dawley (SD), 6-week-old and pecific-pathogen-free rats weighing each 200–230 g, were purchased from Theodor Bilharz Research Institute (Cairo, Egypt) and were housed at Animal Care Center, Cairo University, with an air-condition (22 ±1°C, 55% humidity, and 12:12 hrs daylight/darkness cycles), and a free access to standard laboratory feed and water, according to the study protocol. The study protocol was approved by the Institutional Animal Care and Use Committee (IACUC), Faculty of Science, Cairo University, Egypt. All experimental procedures were carried out in accordance with international guidelines for care and use of laboratory animals.

Rats were randomly divided into six groups with five rats per group. Five rats were considered as the normal control group. Liver fibrosis was induced by intraperitoneally (i.p.) injecting 23 rats with CCl_4_ (1 ml/kg; Sigma-Aldrich, Saint Louis, Missouri, USA) dissolved in paraffin oil, twice a week for 6 weeks (12 doses) [32]. vehicle control rats (n = 5) were injected with an equal volume of paraffin oil alone. 5 rats from the fibrosis group (fibrosis control group), and all rats from normal control groups were euthanized immediately after the last dose (12^th^ dose) of CCl_4_ administration. In the remaining 15 rats of the liver fibrosis group, BM-MSCs (3×10^6^ cells) were injected via the tail vein after 24 hrs of the last dose of CCl_4_ (12^th^ dose). The animals in the treated group were sacrificed at 2, 4, and 6 weeks post-transplantation.

In parallel with the treated group, three CCl_4_- induced rats were injected with saline instead of BM-MSCs, as a recovery group (R-6W) for six weeks later and sacrificed by the end of the study.

### Histopathology and Sirius Red stain

To investigate liver damage in CCl_4_ and in CCl_4_/MSCs treated rats, a part of the liver tissue was fixed in 10% formyl saline, then embedded in paraffin blocks. Tissue sections (4 μm thick) were stained with hematoxylin and eosin (H&E) for examination by a pathologist as previously described [33].

Sirius Red stain was applied to assess collagen deposition, nuclei were stained with hematoxylin (Sigma-Aldrich, Saint Louis, Missouri, USA), then Picro-Sirius Red stain as per the manufacturer’s instructions (Direct Red 80, Sigma-Aldrich, Saint Louis, Missouri, USA)

### Biochemical analysis of liver functions

Alanine aminotransferase (ALT), aspartate aminotransferase (AST), and alkaline phosphatase (ALP) were measured to assess liver function using commercially available kits (Biomed Diagnostic, Badr city, Egypt) according to manufacturer’s instructions.

### Quantitative real-time reverse transcription polymerase chain reaction (RT-qPCR)

RNA was extracted from liver tissue using RNeasy Mini Kit (Qiagen, Hilden, Germany) according to manufacturer’s instructions. One μg of total RNA was converted into cDNA using cDNA synthesis kit (Qiagen, Hilden, Germany) as per the instructions manual. RT-qPCR was performed according to the instructions of QuantiFast SYBR Green PCR Kit’s manual (Qiagen, Hilden, Germany) to amplify *collagene1α1 (Col1a1)*, *α-fetoprotein* (*Afp*), *Albumin* (*Alb*), *Il17a*, *Il17ra*, *Il17f*, *Il17rc* and *Stat3* genes. All primers’ sequences (Invtrogen, Carlsbad, CA, USA) are mentioned in Table 1.

**Table 1:** Sequence of primers used in RT-qPCR analysis.

Gene	Forward primer	Reverse primer	Accession Number
***Il17a***	CCGAGATAACTTTGAGGCATA	AACGAGGTTTGACTTTCACA	NM001106897.1
***Il17f***	GGAAAAGCCTCCTTTGATCC	ACGGAGCTTCAAGGATGTTG	NM001015011.2
***Il17ra***	GGGTGTATGGCCTCATCAC	ACAGGCAGTGATCAGGAACT	NM001107883.2
***Il17rc***	GACCTCAGAACATTACTTTAAACCACACT	GCCAGAAGCTGGTCCTAACAGA	NM001170565.1
***Col1a1***	GTGCGATGGCGTGCTATGC	CTATGACTTCTGCGTCTGGTGATAC	NM053304.1
***Alb***	GATGCCGTGAAAGAGAAAGC	CGTGACAGCACTCCTTGTTG	NM134326.2
***Afp***	TCTGAAACGCCATCGAAATGCC	AATGTAAATGTCGGCCAGTCCCT	NM012493.2
***Stat3***	CACCCTGAAGCTGACCCAG	TATTGCTGCAGGTCGTTGGT	NM012747.2
***Stat5a***	GCCCTCAGGCTCACTACAAC	AAAGGCGGGGGTCAAGACT	NM017064.1
***Gapdh***	GTATCGGACGCCTGGTTAC	CTTGCCGTGGGTAGAGTCAT	NM017008.4

Real-time PCR was performed using StepOnePlus system (Applied Biosystems, Foster city, CA, USA) and PCR was carried out in 25 μl reaction volume and reaction conditions for amplification of target genes were as follows: initial denaturation at 95°C for 10 min followed by 40 cycles of denaturation at 95°C for 15 s, annealing at 56–60°C for 30 s, and extension at 60°C for 30 s. A melting-curve was performed from 60°C to 95°C reading every 0.3°C with 1s hold between reads. Samples were performed in duplicates and the expression of mRNA was normalized to Gapdh gene as endogenous control and the relative fold difference in expression was calculated using ΔΔCt.

### Immunohistochemistry

Immunohistochemical examination of IL17A and IL17RA was performed using 5 μm paraffin sections. Sections were deparaffinized in xylene and rehydrated in alcohol and distilled water. Retrieval of antigens was performed by heating sections in solution (10 mM citrate buffer, pH 6.0) in a pressure cooker, 3% H_2_O_2_ was used to eliminate endogenous peroxidase. Slides were washed with TBS-Triton three times and one time with TBS for 5 min. Nonspecific binding was blocked using 5% goat serum for 30 min and the blocking buffer was then removed and sections were incubated with a polyclonal anti–IL17A antibody (1:2000) (USBiological, Salem, MA, USA), polyclonal anti–IL17RA antibody (2.5μg/ml) (GeneTex, Irvine, CA, USA) and polyclonal antibody to PCNA (2.5mg/ml) (ThermoFisher Scientific, Rockford, IL, USA). Negative control samples were incubated with rabbit serum replacing first antibody. After incubation overnight at 4°C and washing, the sections were incubated with biotinylated goat anti-rabbit IgG antibody. After washing, peroxidase-coupled antibody was applied for 30 min at room temperature. Bounded antibodies were detected via 3, 3′-diaminobenzidine tetrachloride (Sigma-Aldrich, Saint Louis, Missouri, USA). All sections were then counterstained with hematoxylin. Brown-yellow staining was recognized as positive in the cells.

### Western blot assays

The levels of p-STAT3, p-STAT5, p-SMAD3, and TGFβR2 proteins were assessed in liver tissue as previously described [34]. The liver tissue was homogenized in RIPA buffer containing protease and phosphatase inhibitor cocktail (BioBasic, Ontario, Canada). The total protein levels were quantified using the Biorad assay, 20–30 μg of total protein from the cell lysate were fractionated by sodium dodecyl sulfate polyacrylamide gel electrophoresis (SDS-PAGE) and transferred to a polyvinylidene difluoride (PVDF) membrane (Bio-Rad, Hercules, CA, USA). Membranes were blotted overnight at 4°C with the primary antibodies, anti p-STAT3, anti p-STAT5, anti p-SMAD3 or anti TGFβR2 (ThermoFisher Scientific, Rockford, IL, USA) at concentration (1:1000), and then incubated with the horseradish peroxidase (HRP)-conjugated secondary antibodies against rabbit IgG (ThermoFisher Scientific, Rockford, IL, USA) for 1 hr at room temperature. β-actin was used as loading control. Band intensity was analyzed by ChemiDoc imaging system with Image Lab software version 5.1 (Bio-Rad Laboratories Inc., Hercules, CA, USA).

### Estimation of IL2, IL6 and IL17A protein levels by ELISA

The serum levels of IL17A (Invitrogen, Carls, CA, USA), IL6 and IL2 (KomaBiotech, Seoul, Korea) were measured according to manufacturer’s instructions.

### Statistical analysis

Results were analyzed using statistical package for the social sciences software (SPSS, Chicago, IL, USA), version 24 & 25. Data were expressed as mean ± SD. Comparisons between groups were assessed using analysis of variance (ANOVA) with multiple comparisons post hoc test. P-value less than 0.05 was considered statistically significant.

## Results

### MSCs culture detection and homing

**Fig 1:**
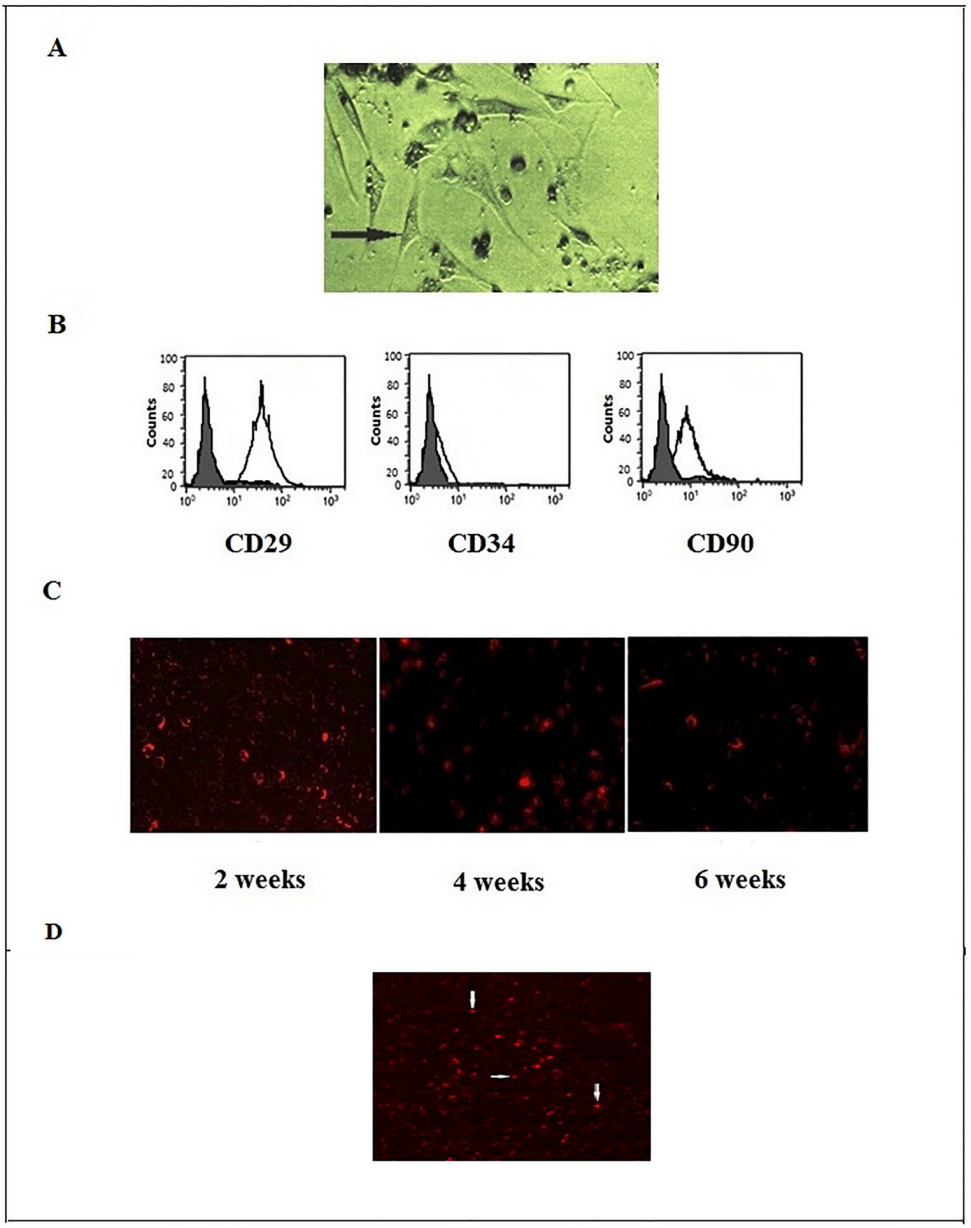
Bone marrow-derived mesenchymal stem cell characterization and homing. **a**. BM-MSCs at 3^rd^ passage displayed fibroblast-like morphology (200X). **b**. Phenotyping of the BM-MSCs analyzed by "flow cytometry" indicated that cells were negative for CD34 and positive for CD90 and CD29 antibodies. **c**. PKH26-labeled BM-MSCs were tracked in liver tissue after 2, 4 and 6 weeks of injection (200X). d. labeled BM-MSCS (red) located near hepatic sinusoid (white arrow).

Successful MSCs were confirmed by their morphology under microscope (Fig 1a) and flow cytometric analysis showed that the surface markers were positive for CD29 and CD90 and negative for CD34 (Fig 1b). After intravenous injection of labeled stem cells in the tail vein of the rats, BM-MSCs were detected in paraffin sections of liver tissue by fluorescent microscope in the treated groups (Fig 1c and 1d).

### BM-MSCs ameliorate liver fibrosis in CCl_4_-induced rats

Serum levels of ALT, AST and ALP are considered as sensitive indicators of liver damage and hepatic function [35]. Therefore, ALT, AST and ALP levels were assessed by colorimetric and kinetic methods and were significantly increased (*P<0*.*05*) in CCl_4_ group by 4, 3 and 2.5 folds, respectively, compared to control groups, while they were significantly decreased (*P<0*.*05*) after treatment with BM-MSCs (Table 2).

**Table 2:** Estimation of liver functions.

	Normal control	Paraffin/oil control	CCl_4_	CCl_4_+BM-MSCs (2w)	CCL_4_+BM-MSCs (4w)	CCl_4_+BM-MSCs (6w)
**ALT (u/l)**	***16*.*00***±***3*.*16***	***16*.*40***±***3*.*36***	***78*.*60***±***15*.*01***	***35*.*00***±***4*.*30***	***33*.*00***±***10*.*93***	***26*.*80***±***6*.*69***
**AST (u/l)**	***11*.*80***±***1*.*79***	***13*.*20***±***2*.*59***	***45*.*60***±***12*.*66***	***32*.*80***±***3*.*42***	***29*.*20***±***10*.*66***	***23*.*00***±***6*.*52***
**ALP (u/l)**	***128*.*20***±***7*.*40***	***128*.*00***±***11*.*25***	***317*.*20***±***30*.*65***	***224*.*40***±***17*.*16***	***198*.*80***±***27*.*65***	***175*.*20***±***29*.*54***

Data were expressed as mean ± SD.

**Fig 2:**
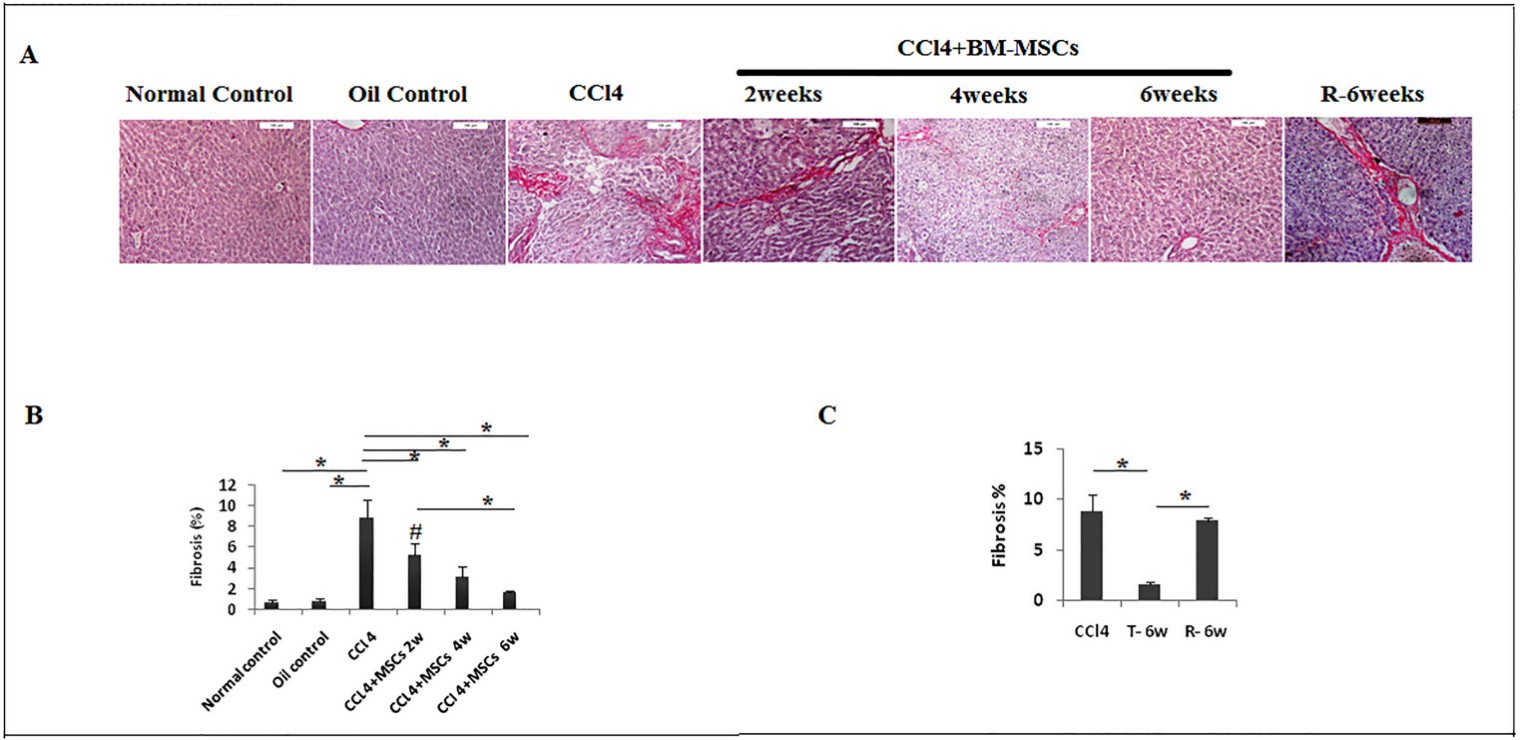
Reduction of fibrosis in injured liver treated with BM-MSCs. a. Sections of paraffin-embedded liver tissue stained with Sirius-red (200X). b. & c. Graphical presentation of collagen levels. Data are expressed as Means (n = 3)± SD. *P<0.05. #*P*<0.05 versus control groups.

The assessment of collagen deposition was provided by Sirius-red stain (Fig 2a), a significant reduction of fibrosis (*P<0*.*05*) was noticed (5.24±1.14%, 3.22±0.98%, and 1.65±0.21%at the 2^nd^, 4^th^ and 6^th^ weeks, respectively) compared to CCl_4_ group (8.82±1.68%) and normal group (0.68±0.21%) as well as oil control group (0.78±0.32%) (Fig 2b). Also, no significant association was detected (*P > 0*.*05*) between recovery group (8.82±1.68%) and CCl_4_ group (7.9±0.36%) (Fig 2c).

**Fig 3:**
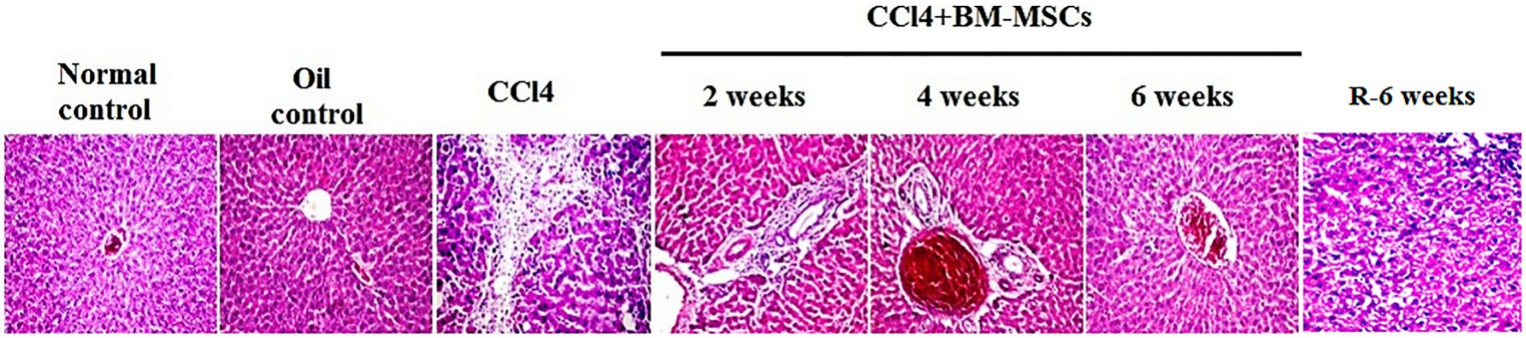
Sections of paraffin-embedded liver tissue stained with H&E, exhibiting histological structure in different study groups (40X).

These results were in accordance with the improvement of histopathological characteristics of fibrotic liver tissue (Fig 3).

### Liver regeneration

**Fig 4:**
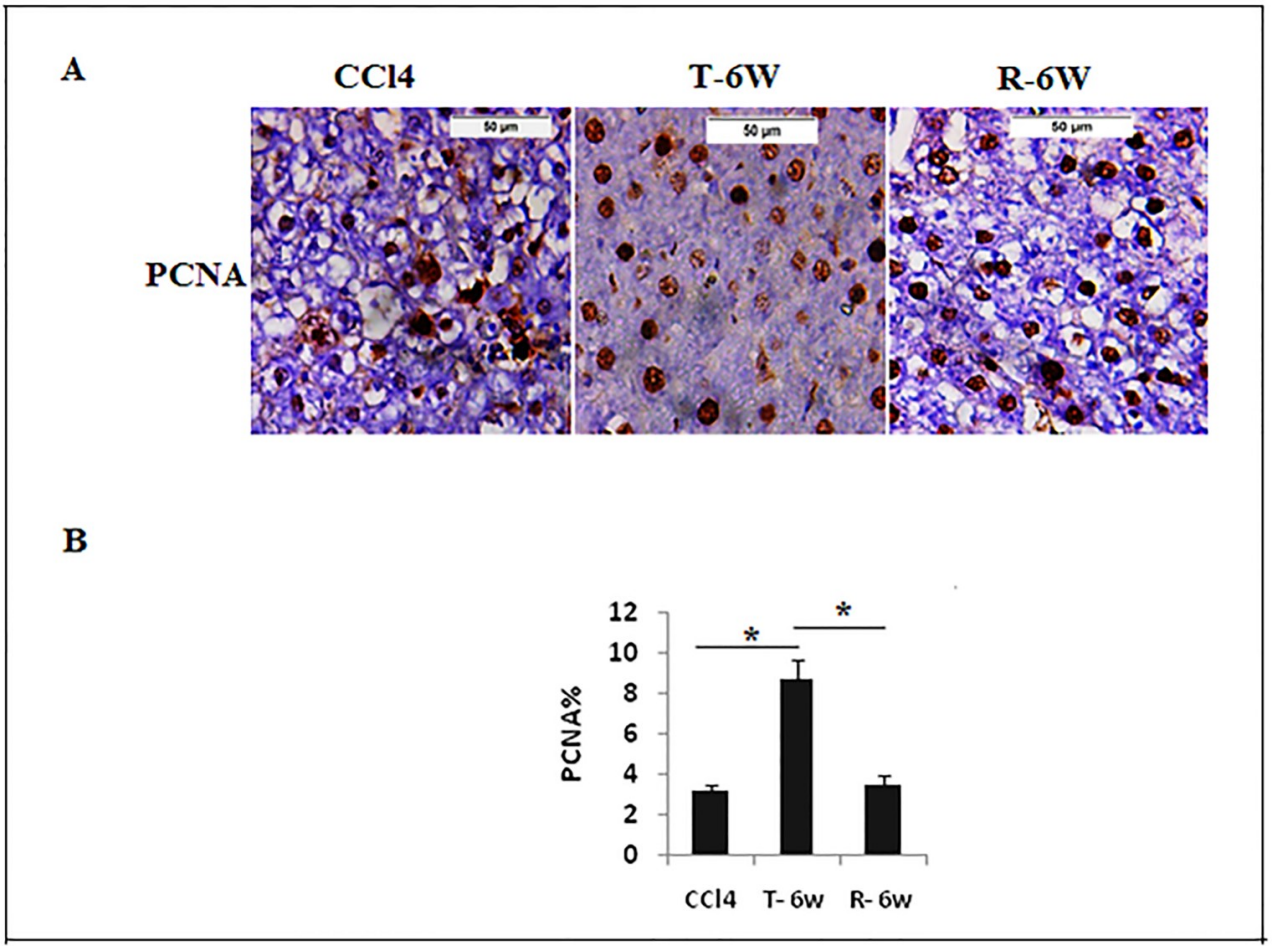
Proliferating nuclear cell antigen protein expression assessesment by immunohistochemistry. a. PCNA expression in liver tissue in CCl_4_, T-6W (BMSCs/6weeks), and R-6W (recovery) groups (Brown). b. quantitative analysis of PCNA expression. Data are expressed as means (n = 3)± SD. (400x).

Enhancement of liver regeneration after transplantation was assessed by measuring PCNA protein expression in the liver tissue (Fig 4a). The 6^th^ week BM-MCs treated group (T-6W) have shown significant elevation of PCNA protein (*P>0*.*05*) compared to CCl_4_ model group and no significant difference (*P>0*.*05*) was detected in CCl_4_ compared to the recovery group (R-6W) (Fig 4b).

**Fig 5:**
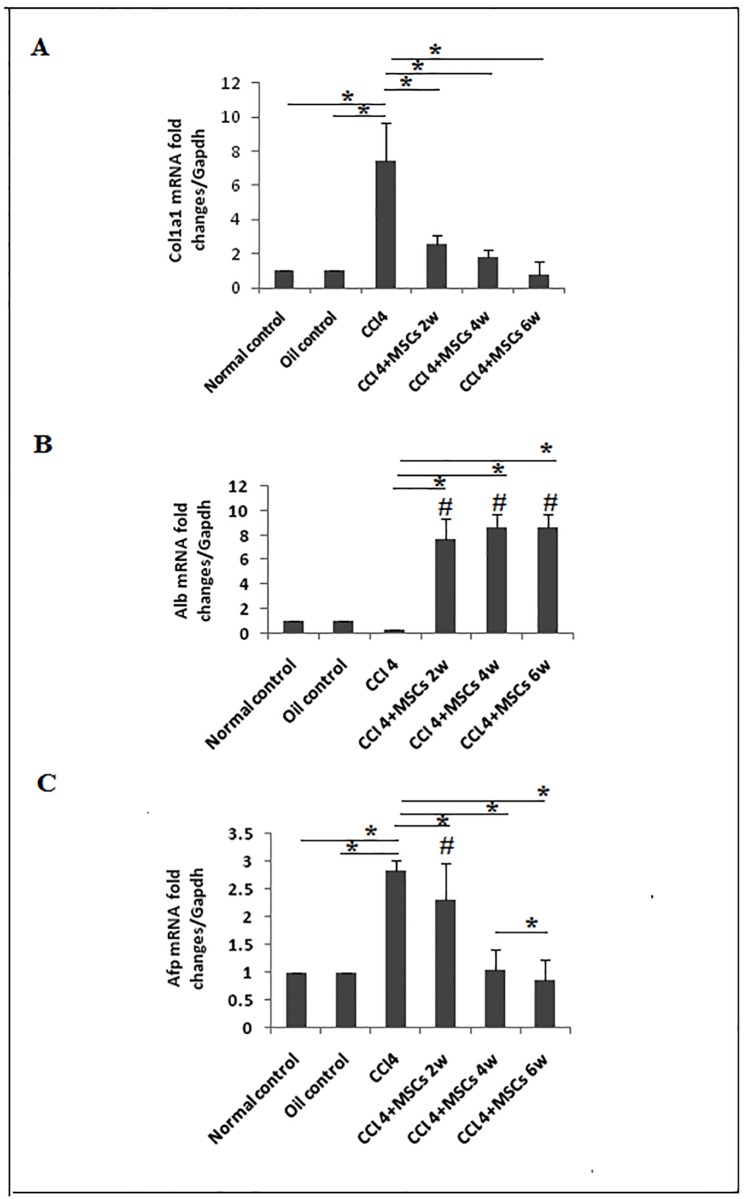
Gene expression of *Alb*, *Afp and Col1a1* in liver tissue. The relative quantification of **a**. *Col1a1*, **b**. *Alb*, *and* **c**. *Afp* were carried out utilizing the 2^–ΔΔCt^ ± standard deviation (SD) method and Gapdh gene expression as housekeeping gene. Each experiment was performed in duplicate and results are expressed as fold induction of mRNA expression (n = 5). *P<0.05. #*P*<0.05 compared to control groups.

As noted earlier, the expression of COL1A1, AFP and ALB are correlated with progression of liver fibrosis [36, 37]. Thus, we assessed the mRNA expression of *Col1a1* and it was significantly upregulated (*P < 0*.*05*) by 7.48 folds in CCl_4_ model group compared to control group, administration of BM-MSCs revealed a significant downregulation of *Col1a1* (*P < 0*.*05*) at the end the 2^nd^, 4^th^ and 6^th^ weeks by 4.9, 5.64 and 7.08 folds, respectively (Fig 5a). CCl_4_-induced group exhibited low *Alb* gene expression which decreased by 0.71 fold compared to both control groups. In response to BM-MSCs administration, *Alb* expression was significantly increased (*P<0*.*05*) in the second, fourth and sixth week groups compared to CCl_4_ group by 7.41, 8.32 and 8.32 folds, respectively (Fig 5b). Moreover, AFP gene expression significantly increased (*P < 0*.*05)* by 1.84 folds in CCl_4_ model compared to the normal diet and paraffin control groups, while following stem cell administration, the expression was significantly reduced (*P < 0*.*05*) by 0.54,1.08 and 1.99 folds at the end of the 2^nd^, 4^th^ and 6^th^ weeks, respectively (Fig 5c).

### BM-MSCs administration downregulated the expression of *IL17A*, *IL17RA* and affected the downstream IL6/STAT3 signaling pathway

**Fig 6:**
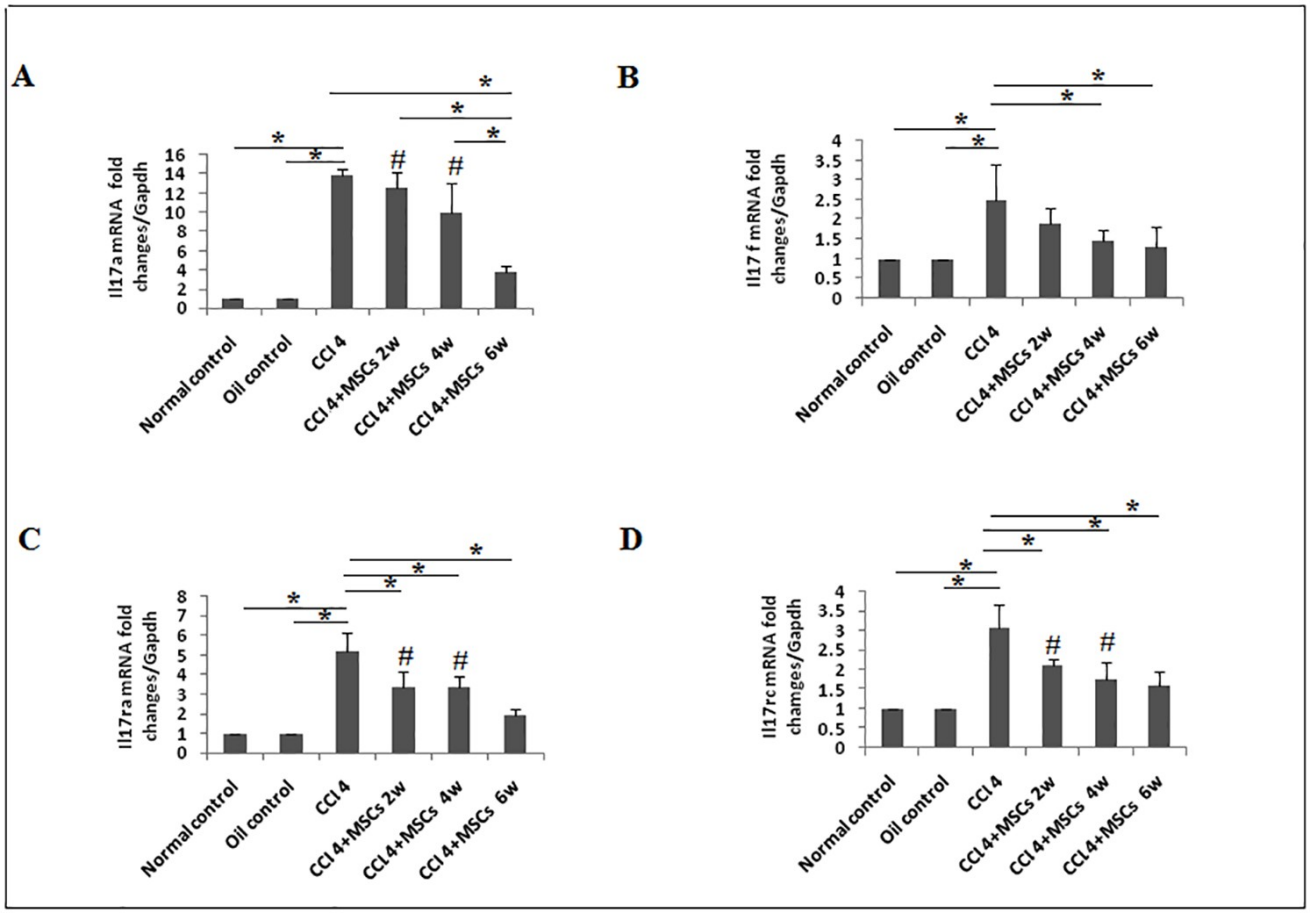
Expression of *Il17a*, *Il17f*, *Il17ra*, and *Il17rc* genes in liver tissue. The data are presented as fold induction of mRNA expression of **a**. *Il17a*, **b**. *Il17f*, **c**. *Il17ra*, and **d**. *Il17rc* in CCl_4_/untreated (n = 5) rats compared to MSCs transplanted (CCl_4_ +BM-MSCs) groups (n = 5) after 2, 4 and 6 weeks. All groups are compared with normal/diet control and oil/vehicle control rats (n = 5). Each experiment was performed in duplicates. Representative data are shown as mean±SD. *P<0.05. #*P*<0.05 compared to control groups.

The critical role of IL-17A signaling in the pathogenesis of liver fibrosis was previously suggested [16, 38]. In the same context, our results showed that mRNA expression of *Il17a*, *Il17ra*, *Il17rc* and *Il17f* were significantly overexpressed (*P < 0*.*05*) by 12.81, 4.2, 2.08 and 1.5 folds, respectively in CCl_4_ model group compared to control groups (Fig 6a–6d). BM-MSCs administration ameliorated the expression of *Il17a* by 1.32, 3.8 and 9.99 folds at the end of the 2^nd^, 4^th^ and 6^th^ weeks, respectively, with a significant reduction (*P < 0*.*05*) observed only by week six of administration compared to CCl_4_ group (Fig 6a). *Il17f* gene was downregulated by 0.62 fold in 2^nd^ week and reduced significantly (*P < 0*.*05*) by 1.02 and 1.19 folds at the end of the 4^th^ and 6^th^ weeks, respectively, compared to the CCl_4_ model (Fig 6b). The expression of *Il17ra* mRNA was significantly downregulated (*P <0*.*05*) compared to CCl_4_ group by 1.81, 1.87 and 3.32 folds at the 2^nd^, 4^th^ and 6^th^ weeks, respectively (Fig 6c). *Il17rc* gene expression was significantly reduced (*P<0*.*05*) after BM-MSCs treatment by 0.95, 1.34 and 1.49 folds at the end of the 2^nd^, 4^th^ and 6^th^ weeks, respectively (Fig 6d).

**Fig 7:**
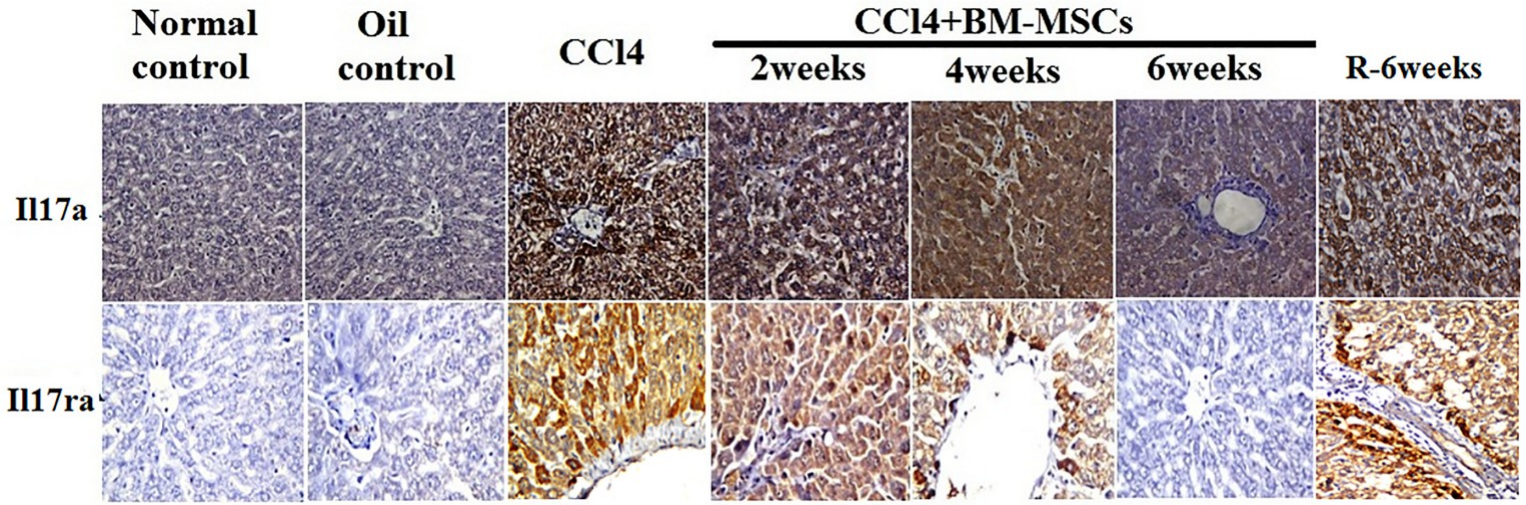
IL17A and IL17RA protein expression in liver tissue evaluated by IHC method. Upper panels are representatives of IL17 expression and it is noted that it was very weak in normal and paraffin/oil control group, severe in CCl_4_/fibrosis and recovery groups, high in CCl_4_+BM-MSCs/2 weeks group, moderate in CCl_4_+BM-MSCs/4weeks group and mild CCl_4_+BM-MSCs/6 weeks group. Lower panels are representatives of IL17RA expression and it is noted that it was absent in normal and paraffin/oil control group, high in CCl_4_/fibrosis and recovery groups, moderate in CCl_4_+BM-MSCs/2weeks group, mild in CCl_4_+BM-MSCs/4weeks group and nil in CCl_4_+BM-MSCs/6 weeks group (400X).

Similar results were observed at the protein level, where IL17A protein was almost absent in normal and paraffin oil control groups of liver tissue while the tissue derived from CCl_4_-induced fibrosis group and recovery group showed high degree of expression of IL-17A and IL17RA proteins. After MSCs transplantation, the tissue exhibited a gradual decrease of the expressed IL17A and IL17RA proteins in accordance with the times of treated groups (Fig 7). Immunoreactivity was localized intracellulary in the hepatocytes surrounding the central vein portal area.

**Fig 8:**
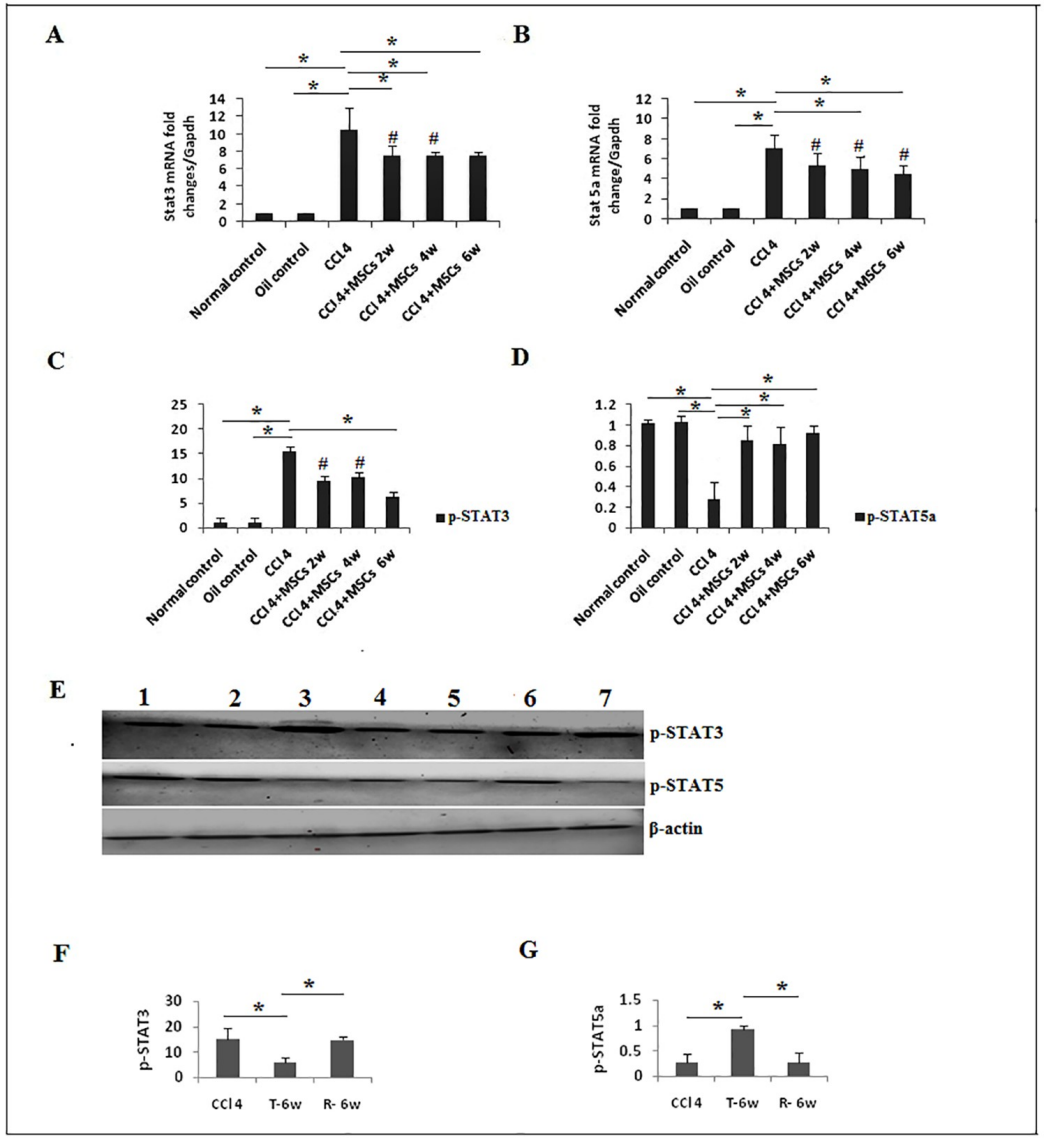
Gene and protein expression of STAT3 and STAT5. **a**. *Stat3* and **b**. *Stat5a* gene expression was measured by qRT-PCR (n = 5). **c**. p-STAT3, and **d**. p-STAT5 protein analysis, representative data of western blot are shown as mean±SD. *P<0.05. #*P*<0.05 compared to control groups. **e**. Expression of STAT3, and STAT5 proteins in liver tissues (n = 3) was assessed by western blot (**WB**) analysis. Quantative expression analysis of f. p-STAT3. **g**. p-STAT5a in. **1**. Normal control. **2**. Oil control. **3**. CCl_4_ groups.**4**. CCl_4_+BM-MSCs/2 weeks. **5**. CCl_4_+ BM-MSCs/4 weeks. **6**. CCl_4_+MSCs/6 weeks. 7. Recovery group (R-6W).

Noticeably, one of the Stat3 signaling cytokine with pro-fibrogenic properties is IL17A, enhancing activation of hepatic myofibroblasts either directly or through IL6 secretion [16, 39] and collected evidences indicate the contribution of activated STAT3 in liver fibrosis [18]. Thus, we assessed the expression levels of STAT3 and our CCl_4_ model group revealed that *Stat3* mRNA was significantly overexpressed (*P <0*.*05*) by 9.45 folds compared to control groups. *Stat3* mRNA was significantly (*P<0*.*05*) reduced by 2.85, 2.9 and 2.9 folds after the 2^nd^, 4^th^ and 6^th^ weeks, respectively, after treatment with BM-MSCs (Fig 8a). We analyzed p-STAT3 levels in both control groups, CCl_4_ group and BM-MSCs treated groups. Phosphorylated STAT3 levels were significantly (*P<0*.*05*) upregulated in the CCl_4_ group compared to both controls, while significant (*P<0*.*05*) reduction of p-STAT3 was observed after week six of BM-MSCs administration (Fig 8c and 8e). No significant difference in of p-STAT3 expression was noticed between CCl_4_ and recovery groups (*P>0*.*05*) (Fig 8f).

**Fig 9:**
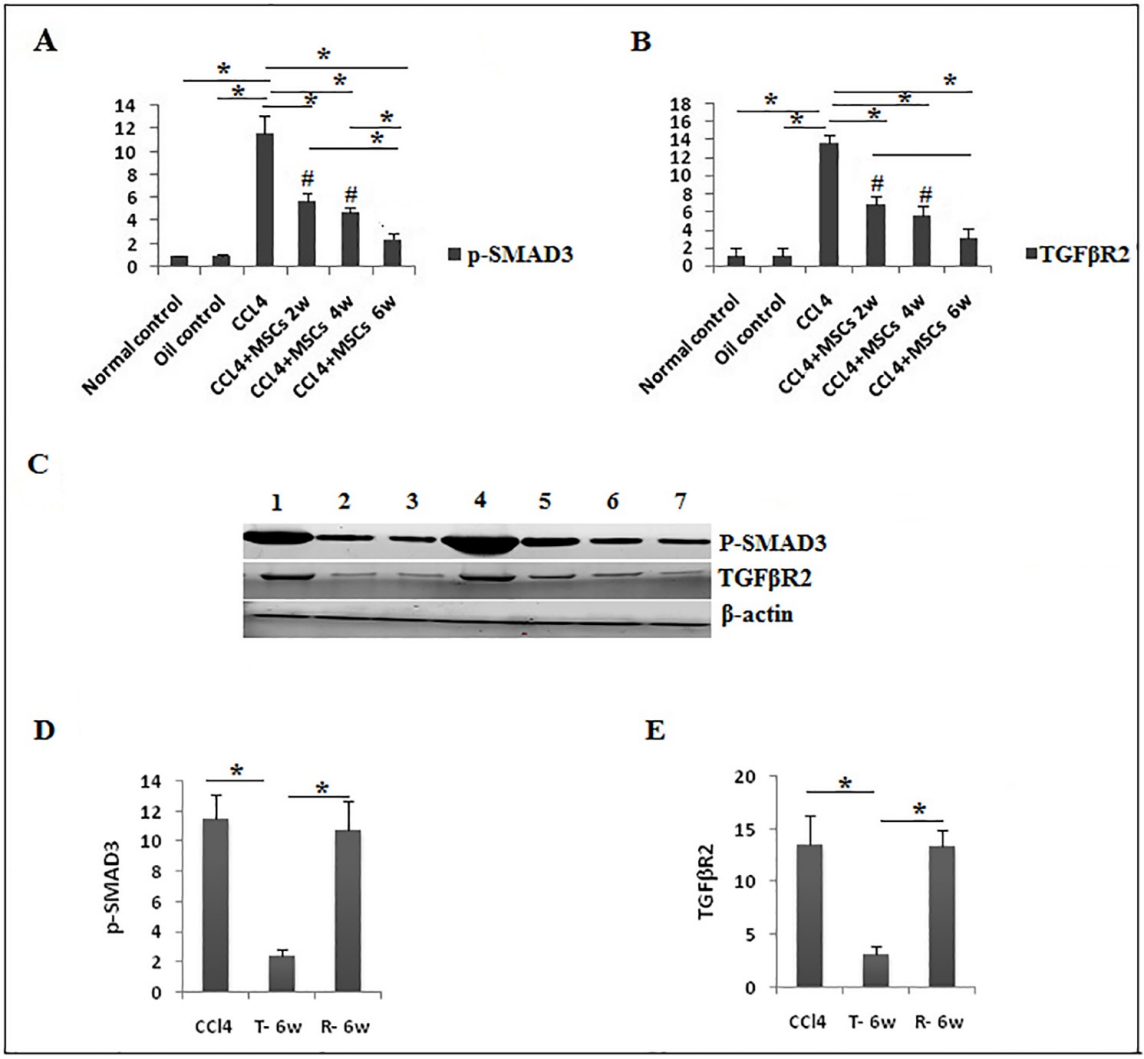
Protein expression of p-SMAD3 and TGFβR2. **a**. p-SMAD3, and **b**. TGFβR2 protein analysis, representative data of western blot are shown as mean±SD in different groups (n = 3). **c**. Expression of p-SMAD3 and TGFβR2 proteins by western blot **(WB)** in liver tissues. Quantative expression analysis of .d. p-SMAD3. e. TGFβR2 **in 1**. Recovery group (R-6W).**2**. Normal control. **3**. Oil control. **4**. CCl_4_ groups. **5**. CCl_4_+BM-MSCs/2 weeks. **6**. CCl_4_+ BM-MSCs/4 weeks. **7**. CCl_4_+MSCs/6 weeks.

The molecular consequences of p-SMAD3 and TGFβR2 proteins and development of liver fibrosis were demonstrated that Stat3 is essential to collaborate with SMAD3 mediating TGFβ induced fibrotic response in hepatic stellate cells [40]. In correlation with our data, p-SMAD3 (Fig 9a and 9c), and TGFβR2 (Fig 9b and 9c) proteins significantly increased (*P<0*.*05*) in the fibrosis group compared to controls, and they were significantly downregulated (*P<0*.*05*) from the 2^nd^ week of BM-MSCs treatment. On the other hand, CCl_4_ and recovery group showed no significant difference (*P>0*.*05*) in expression of p-SMAD3 and TGFβR2 (Fig 9d and 9e).

**Fig 10:**
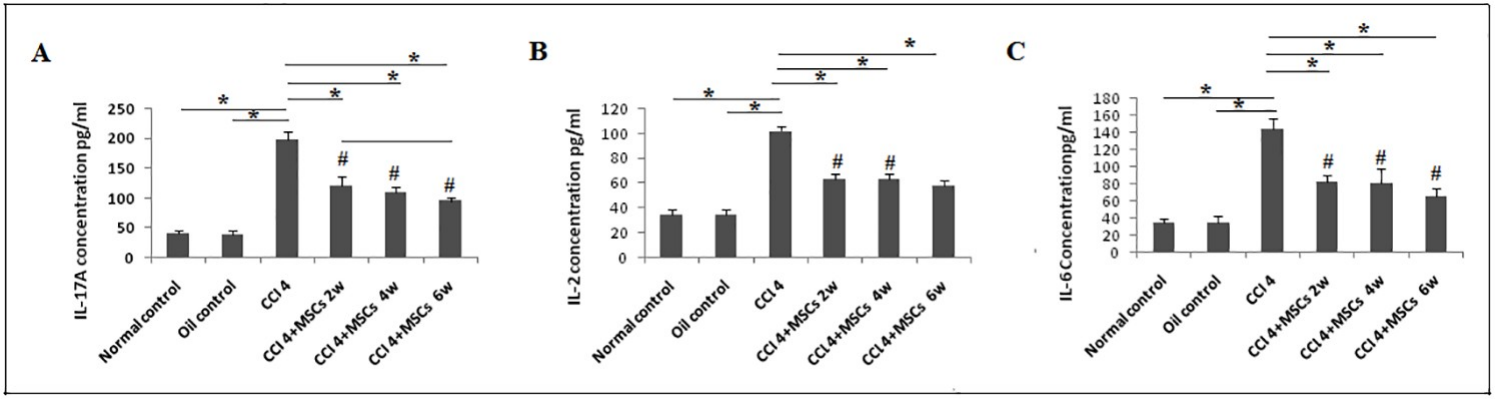
IL17A, IL6 and IL2 protein expression levels in serum measured by ELISA. The data shown in (**a**), (**b**) and (**c**) are presented as the mean ±SD *P <0.*05*. #*P*< 0.05 compared to control groups.

In harmony with these data, a significant increase of IL17A serum protein was observed in the untreated CCl_4_ group while a significant reduction was observed along the 6 weeks of BM-MSCs treatment (Fig 10a). Moreover, our results displayed a significant elevation of IL6 (*P<0*.*05*) in serum of CCl_4_ group with a significant reduction (*P < 0*.*05*) in all treated groups (Fig 10c).

### BM-MSCs administration affects the expression of *Stat5a* gene and p-STAT5A

In response to liver injury, the anti-fibrotic effect of Stat5 was demonstrated in various models of liver fibrosis [41]. Our results showed that mRNA of *Stat5a* was significantly upregulated (*P<0*.*05*) by 6.06 folds in CCl_4_ group, but mRNA *Stat5*a was reduced by 1.72 folds after the 2^nd^ week of treatment, significant downregulations (*P<0*.*05*) were observed by 2.08 and 2.57 folds at 4^th^ and 6^th^ weeks, respectively (Fig 8b). Interesting data were obtained by the activated form of STAT5 protein, where a significant reduction of p-STAT5 protein (*P<0*.*05*) was noticed in CCl_4_-induced group and significant elevation was observed by the 2^nd^ week in response to BM-MSCs treatment (Fig 8d and 8e),.CCl_4_ and recovery groups showed no significant difference (*P>0*.*05*) in p-STAT5 protein expression (Fig 8g). An early investigation described the lack of IL2 or disruption of its signaling through deletion of Stat5 resulted in enhanced Th17 cell differentiation [42]. Here, our CCl_4_ group showed a significant elevation of IL2 protein (*P<0*.*05*) in serum and a significant reduction (*P<0*.*05*) in response to BM-MSCs administration (Fig 10b).

## Discussion

Recent studies illustrated the crucial role that IL17A plays in the development and progression of hepatic fibrosis [14], along with the anti-fibrotic activity of BM-MSCs [12, 43]. In this study, we demonstrated a therapeutic potential of BM-MSCs in CCl_4_-induced rat liver fibrosis through inhibition of IL17A/F isoforms expressed genes and IL17 associated signaling pathway.

The current study represents the first report that highlights the inhibitory effect of BM-MSCs on IL6/STAT3 pathway in liver fibrosis. Previous studies have discussed the correlation between MSCs and IL6 in the progression of cancer [40, 44]. One study reported the secretion of IL6 by the BM-MSCs which activated IL6/Stat3 pathway promoting the invasion of the hepatocellular carcinoma cell line [30]. A recent study investigated the role of MSCs at different stages of hepatocellular carcinoma as well as liver fibrosis in rats [44]. In that study, the early stages of hepatocellular carcinoma showed the ability of MSCs to suppress liver cancer and reduce liver fibrosis and to employ anti-inflammatory effect via IL6 inhibition. On the other hand, MSCs promoted cancer in progressive stages due to the elevation of proinflammatory cytokines (IFNγ, TNFα, IL6 and IL1β) expression in the tumor microenvironment [44]. These results indicate that the role of BM-MSCs could be perplexing according to the surrounding microenvironment.

In the present study, in parallel to the improvement of the histopathological structure of liver tissue confirming the therapeutic effect of BM-MSCs, the liver biomarkers ALT, AST, and ALP levels in the blood were significantly recovered and the fibrogenic *Col1a1* mRNA were gradually decreased in the treated group and reached normal levels after 6 weeks of BM-MSCs implantation. These results agree with the previous studies reporting the ability of BM-MSCs to repair CCl_4_-damaged liver by reduction of inflammation, collagen deposition, and remodeling [11, 12, 45]. The protective function of MSCs in liver fibrosis as well as liver inflammation could be explained by the modulatory effect of MSCs via paracrine mechanisms on reducing the function of activated hepatic stellate cells (HSCs) through secretion of IL-10. In addition, MSCs promote apoptosis of HSCs via hepatocyte growth factor (HGF) [46].

Upon BM-MSCs treatment, the mRNA levels of the unique hepatocyte marker Alb increased, while the Afp mRNA level decreased in a time dependent manner and these results agree with a previous study that correlated these changes in expression to the differentiation of BM-MSCs into hepatocyte-like cells and normalization of Alb could possibly need more time [47].

IL17 signaling contributes to the pathogenesis of liver fibrosis [16]. We evaluated the effect of BM-MSCs administration to CCl_4_-induced liver fibrosis group on the expression of il17a/f isoforms and their receptors' (*Il17a and Il17r*) genes. Here, upregulation of the expressed *Il17a*, *Il17f*, *Il17ra* and *Il17rc* were detected as previously reported [48]. Consequent to BM-MSCs administration, all genes were almost normalized after 6 weeks. Also, we reported elevation of IL17A and IL17RA proteins in fibrotic liver tissue with their reduction in response to BM-MSCs treatment. This could be anticipated to the anti-inflammatory modulation of MSCs [49] and its role in reduction of Th17 cells in liver [50].

Hence, IL17 is associated with IL6 concentration [51, 52] and serum level of IL6 and IL17 evaluate the severity of liver fibrosis [53, 54]. To our knowledge, the effect of BM-MSCs on the downstream pathway IL6/STAT3 has not been previously discussed. In this study, the anti-inflammatory role of MSCs was suggested as our results revealed that the upregulated IL6 and IL17A serum proteins in the fibrosis model were downregulated gradually with the improvement of fibrosis after BM-MSCs administration which agrees with a previous report [55].

The role of STAT3 is controversial in mediating signaling of liver fibrosis [18, 26]. On the other hand, IL17A signaling activates STAT3 and is suggested to promote the development of liver fibrosis [13]. Here, we demonstrated that upregulations of Phosphorylated STAT3 and *Stat3* gene expression in CCl_4_ group were downregulated over time of BM-MSCs transplantation.

Furthermore, STAT3 signaling pathways are essential for IL6-dependent hepatic stellate cells activation [56]. Thus, we suggest that BM-MSCs ameliorate liver fibrosis via downregulation of IL17A dependent IL-6/STAT3 signaling pathway.

SMAD3 is a key of signal transduction pathways in liver fibrosis [22] and elevated STAT3 phosphorylation in fibrosis is combined with TGFβ1 and SMAD3 activation [18, 57]. Otherwise, the increased p-SMAD3 and TGFβR2 expression has been reported in CCl_4_-induced acute liver injury [58] and BM-MSCs led to the recovery of liver function via the TGFβ1/SMAD signaling pathway [34]. Herein, we report the downregulation of p-SMAD3 and TGFβR2 after BM-MSCs administration in rats with liver fibrosis, suggesting the inhibitory effect of BM-MSCs on CCl_4_-induced fibrosis probably by affecting TGFβ/SMAD signaling pathways through reduction of TGFβR2 and phosphorylation of SMAD3.

The antifibrotic effect of STAT5 was demonstrated in murine models of liver fibrosis [59, 60]. Few studies discussed the effect of BM-MSCs on the STAT5 expression. Hence, the current study declared the elevated level of *Stat5a* gene expression in CCl_4_-induced group with gradual reduction over time in response to BM-MSCs administration. In contrast, Phosphorylated Stat5a was downregulated in CCl_4_ group and was augmented after BM-MSCs transplantation.

Even though expression of *Stat3* and *Stat5a* mRNA postulated no variability in CCl_4_ groups, but the activated form gave the answer towards fibrogenesis and treatment. This might be justified by the fact that TGFβ is crucial in the development of liver fibrosis, as previous data proposed that upon development of liver fibrosis, TGFβ traps STAT5 in hepatocytes and lead to the activation of STAT3 [61]. This might verify the role of BM-MSCs in reduction of TGFβ1 [62]. Consequently, the activated form of STAT5A increased as well as fibrosis decreased in response to treatment.

It is crucial to mention that the recovery group in our study did not show any amelioration of fibrogenesis or studied parameters after 6 weeks of recovery and our results are in accordance with previous studies [11, 43, 47, 63–66]

In conclusion, we have detected BM-MSCs in the recipient rats suggesting their role in ameliorating liver tissue damage through their immunoregulatory activities by suppressing the inflammatory genes IL17A/RA and downregulating the IL6/STAT3 pathway.
